# Draft Genome Sequences of *Bacillus* and *Paenibacillus* Species Isolated from Seeds of Citrullus lanata (Watermelon), Cucurbita moschata (Butternut Squash), and Cucurbita pepo L. var. *pepo* L. (Pumpkin)

**DOI:** 10.1128/MRA.00727-20

**Published:** 2020-08-20

**Authors:** Eman M. Khalaf, Manish N. Raizada

**Affiliations:** a Department of Plant Agriculture, University of Guelph, Guelph, Ontario, Canada; b Department of Microbiology and Immunology, Faculty of Pharmacy, Damanhour University, Damanhour, Egypt; University of Arizona

## Abstract

Here, we announce the draft genome sequences of four endophytic bacilli isolated from surface-sterilized seeds of three cucurbit species, *Bacillus* sp. strains EKM417B and EKM420B (from Citrullus lanata [watermelon]) and EKM501B (from Cucurbita moschata [butternut squash]) and *Paenibacillus* sp. strain EKM301P (from Cucurbita pepo L. var. *pepo* L. [pumpkin]). These strains previously demonstrated biostimulant and biocontrol activities.

## ANNOUNCEMENT

Plant microbiomes have evolved to perform defensive/growth-promoting functions (1–3). 16S Illumina sequencing of cucurbit seeds revealed the dominance of spore-forming, Gram-positive bacteria (e.g., *Bacillus*/*Paenibacillus* genera) (4), consistent with aerobically cultivated microbiota (5). We isolated unique colonies from cultivated surface-sterilized cucurbit seeds. They were identified as *Bacillus* sp. strains EKM417B and EKM420B (from Citrullus lanata [watermelon]), *Bacillus* sp. strain EKM501B (from Cucurbita moschata [butternut squash]), and *Paenibacillus* sp. strain EKM301P (Cucurbita pepo L. var. *pepo* L. [pumpkin]) using 16S rRNA gene primer pair 799F and 1492R and then submitted to GenBank (accession numbers KT281355, KT281357, KT281359, and KT281432, respectively) (5). Since many commercial microbial fertilizers/biocontrol agents are *Bacillus*/*Paenibacillus* based (1, 2), these candidate endophytes were tested for beneficial traits *in vitro*/*in planta* (5, 6). All four strains showed *in vitro* protease activity (5) and acetoin/diacetyl production (volatiles) and suppressed Phytophthora capsici (6). Other *in vitro* traits were scored, albeit inconsistently, as follows: EKM417B/EKM420B displayed pectinase and RNase activities; EKM417B secreted cellulase and reduced the disease severity of Podosphaera fuliginea (foliar fungal pathogen) *in planta*; EKM501B grew on N_2_-free medium, produced indole-3-acetic acid (IAA/auxin) and ribonucleases, and suppressed Rhizoctonia solani; and EKM301P secreted cellulase and antagonized Fusarium graminearum and Rhizoctonia solani (5, 6).

The strains were cultured overnight on LB agar from original −80°C glycerol stocks. Single colonies were inoculated into lysogeny broth (overnight, 37°C, 250 rpm). Genomic DNA was extracted using DNeasy UltraClean microbial kits (Qiagen, catalog number 12224-50) and adjusted to 50 ng/μl. Libraries were constructed using TruSeq DNA Nano library prep kits (KAPA HyperPrep kit, catalog number KK8504) and then sequenced using the Illumina NovaSeq 6000 system to produce 1,461,384 (EKM417B), 1,659,509 (EKM420B), 1,823,824 (EKM501B), and 2,124,086 (EKM301P) raw reads in 150-bp paired-end format. Quality-trimmed reads (with a quality score of 30) were *de novo* assembled using the EvoCAT pipeline (Evogene Clustering and Assembly Toolbox) and identified using KmerFinder 3.2 (7) by conducting a BLAST search against Bacillus velezensis strain KD1 (GenBank accession number NZ_CP014990.2) (EKM417B and EKM420B), Bacillus cereus strain FORC087 (NZ_CP029454.1) (EKM501B), and Paenibacillus polymyxa strain SQR-21 (NZ_CP006872.1) (8) (EKM301P) as the top genome matches (Table 1). The protein predictions were completed using Prodigal (9) and then matched against the NCBI nonredundant protein database using Blastp (10). Peptide domains were identified using InterProScan 5.32-71.0 (11). Default parameters were used for all software unless otherwise specified. The statistics of the genomes are provided in Table 1.

**TABLE 1:** Statistics and accession numbers of the sequenced *Bacillis* and *Paenibacillus* strains

Metrics	Data for bacterial strains^*a*^			
	*B. velezensis* EKM417B	*B. velezensis* EKM420B	B. cereus EKM501B	*P. polymyxa* EKM301P
Genome size (bp)	3,972,727	3,908,635	5,734,326	5,661,237
No. of clean reads	1,228,648	1,426,781	1,613,183	1,755,075
No. of contigs	310	83	105	198
*N*_50_ (bp)	996,524	996,284	239,839	531,804
Maximum scaffold length (bp)	1,083,982	1,084,468	876,274	1,218,405
Minimum scaffold length (bp)	206	206	202	203
Avg genome coverage (×)	93	109	91	90
No. of predicted genes	3,693	3,553	5,055	4,560
G+C content (%)	45	47	36	52
GenBank accession no.	JAAMNY000000000	JAAMNX000000000	JAALFZ000000000	JAALFM000000000
SRA accession no.	SRR11043897	SRR11051662	SRR11051671	SRR11048276

a The taxonomy of bacterial species was obtained from the updated GenBank databases.

Genome mining identified candidate genes involved in biofertilizer/biocontrol metabolic pathways, including those discussed above. These genes encode proteins involved in nitrogen fixation, phytase, alkaline phosphatase, carbon-nitrogen hydrolase, trehalose-6-phophate hydrolase, and tryptophan synthase (IAA/auxin production) (12–14). Biocontrol/systemic resistance elicitor candidate genes encode hydrolytic enzymes (β-glucanase, chitinase, cellulase, proteases, pectin/pectate lyases, lipases, ribonucleases) (14–16), exopolysaccharide synthesis protein (colonization ability) (17), butanediol-dehydrogenase-like (acetoin production) (18), iron-siderophore-like, polyketide synthase, nonribosomal peptide synthase (NRPS) (19), phenazine biosynthesis PhzF protein (except EKM301P) (20), bacteriocins (class IId [EKM417B/EKM420B], class IIb [EKM501B], and thiopeptide-type [EKM301P]) (21), and phenylalanine/histidine ammonia-lyases (except EKM301P) (22). The exception is that EKM501B lacked β-glucanase, pectate lyase, and phytase but encoded aerobactin siderophores (23). In conclusion, cucurbit seeds host bacilli as vectors that encode candidate beneficial traits for plants.

### Data availability.

This whole-genome shotgun project and the Illumina raw reads have been deposited in DDBJ/EMBL/GenBank and the SRA, respectively, under the accession numbers provided in Table 1.
